# Modulation of the tumor microenvironment and mechanism of immunotherapy-based drug resistance in breast cancer

**DOI:** 10.1186/s12943-024-01990-4

**Published:** 2024-05-07

**Authors:** Moumita Kundu, Ramesh Butti, Venketesh K. Panda, Diksha Malhotra, Sumit Das, Tandrima Mitra, Prachi Kapse, Suresh W. Gosavi, Gopal C. Kundu

**Affiliations:** 1 School of Biotechnology, KIIT Deemed to be University, Bhubaneswar, 751024 India; 2 Department of Internal Medicine, Division of Hematology and Oncology, University of Texas Southwestern Medical Center, Dallas, TX 75235 USA; 3 National Centre for Cell Sciences, Savitribai Phule Pune University Campus, Pune, 411007 India; 4 School of Basic Medical Sciences, Savitribai Phule Pune University, Pune, 411007 India; 5 Kalinga Institute of Medical Sciences (KIMS), KIIT Deemed to be University, Bhubaneswar, 751024 India; 6 Present Address: Department of Pharmaceutical Technology, Brainware University, West Bengal, 700125 India

**Keywords:** Breast cancer, Tumor microenvironment, Cancer-associated fibroblast, Tumor-associated macrophage, Immune resistance, Therapeutic approach

## Abstract

Breast cancer, the most frequent female malignancy, is often curable when detected at an early stage. The treatment of metastatic breast cancer is more challenging and may be unresponsive to conventional therapy. Immunotherapy is crucial for treating metastatic breast cancer, but its resistance is a major limitation. The tumor microenvironment (TME) is vital in modulating the immunotherapy response. Various tumor microenvironmental components, such as cancer-associated fibroblasts (CAFs), tumor-associated macrophages (TAMs), and myeloid-derived suppressor cells (MDSCs), are involved in TME modulation to cause immunotherapy resistance. This review highlights the role of stromal cells in modulating the breast tumor microenvironment, including the involvement of CAF-TAM interaction, alteration of tumor metabolism leading to immunotherapy failure, and other latest strategies, including high throughput genomic screening, single-cell and spatial omics techniques for identifying tumor immune genes regulating immunotherapy response. This review emphasizes the therapeutic approach to overcome breast cancer immune resistance through CAF reprogramming, modulation of TAM polarization, tumor metabolism, and genomic alterations.

## Introduction

Breast cancer is the most diagnosed malignancy in females, with about 2.3 million new cases globally in the year 2020, which accounted for 11.7% of all cancer incidences [1]. According to the International Agency for Research on Cancer (IARC), these numbers are estimated to increase to over 3 million by 2040 [1]. The cancer progression is a multistep integrated process controlled by several genetic and epigenetic factors. Some researchers stated that epigenetic alteration is another hallmark of most cancers due to its critical role in the initiation of carcinogenesis [2–5]. However, cancer develops because of a chaotic tumor microenvironment (TME), including various infiltrating immune cells like tumor-associated macrophages (TAMs), dendritic cells (DCs), lymphocytes, and other stromal cells like cancer-associated fibroblasts (CAFs), endothelial cells, pericytes, and extracellular matrix (ECM) [6]. All these components participate in a complex manner through cell-cell and cell-matrix interactions to shape the microenvironment conducive to tumor progression [3, 7, 8]. Cancer cells frequently educate stromal cells, such as fibroblasts, macrophages, vascular cells, adipocytes, and immune cells, to support their growth and spread to distant sites. The stromal component might dominate the tumor tissue in most solid cancers [9]. CAFs constitute a significant part of stroma, and various tumor-derived factors are known to induce the activation of fibroblasts to CAFs [10]. In addition to cancer cells, different infiltrating immune cells like tumor-associated neutrophils (TANs), TAMs, DCs, and mast cells (MCs) have subsequently appeared to enhance the activation of stromal cells, which, in turn, shape the immune suppressive TME. This interplay comprises immune-inhibitory circles to provide a favorable TME for tumor growth. Moreover, CAFs indirectly alter anticancer immunity and induce T cell dysfunction and immunologic tolerance by upregulating the expression of immune checkpoint molecules like PD1/PD-L1 [7]. The dynamic and mutual association between cancer cells and the TME has the potential to either curb or promote the spread of the disease. Immune cells that have invaded the tumors prevent their growth by destroying immunomodulatory neoplastic cells. However, they might also be responsible for developing tumor resistance to treatment by influencing tumor immunogenicity and selecting tumor clones that can cause immune exhaustion [11]. Moreover, the immune cells in the TME have a dual function in cancer development and metastasis. The type 1 helper T cells (Th1), cytotoxic T lymphocytes (CTLs), and natural killer cells (NK cells) are associated with an immune stimulant microenvironment. In contrast, the regulatory cells of the TME, including type 2 helper T cells (Th2), TAMs, regulatory T cells (Tregs), and myeloid-derived suppressor cells (MDSCs), are associated with immunosuppressive microenvironment and poor outcomes [12, 13]. These cells prevent tumor growth by eradicating immunogenic neoplastic cells or altering tumor immunogenicity, aiding tumor escape [14]. Besides these cells, chemokines and cytokines are essential members of the tumor immune microenvironment (TIME) and play a significant role in maintaining the equilibrium between protumor and antitumor immune responses [15]. The intricate interactions between the cancer cells and the immunological niche affect immunotherapy and many other anticancer therapies.

The development of immuno-based therapy in breast cancer has made significant progress over the past two decades. Though different immunotherapeutic strategies have been explored in breast cancer, the number of immunotherapy-based clinical trials increased after the advent of immune checkpoint inhibitors (ICIs) and antibody-drug conjugates (ADCs). As of January 2022, according to the data identified on clinicaltrials.gov, there were 745 immunotherapy-based trials enrolling patients with solid tumors of different cancers, out of which 450 trials (60.4%) were explicitly dedicated to breast cancer [16]. The ongoing development of immunotherapy has contributed to improved outcomes for many breast cancer patients. Nevertheless, insights from clinical landscapes highlight that TIME composition strongly influences the efficacy of immunotherapy [17]. Importantly, immune cells are now recognized as critical players in the emergence of resistance mechanisms to immunotherapy in breast cancer. These mechanisms hinder the establishment of long-lasting treatments and cause cancer growth [11].

This review article focuses on the complex and dynamic function of TME to elucidate the interplay between the stromal and immune cells. It aims to explore therapeutic strategies that may reverse immunotherapy resistance in breast cancer.

## Recent clinical advances in breast cancer immunotherapy

Immunotherapy is a rapidly evolving field in the treatment of cancer. It involves harnessing the body’s immune system to recognize, target, and eliminate cancer cells. Several types of immunotherapy strategies have shown promising results in treating various cancers. These involve developing ICIs including monoclonal antibodies (mAbs) to block the immunosuppressive molecules and to improve the cytotoxicity of tumor-infiltrating lymphocytes such as CTLA4, PD1, and PD-L1. In addition, ADCs and cancer vaccines have also exhibited the potential to deliver cytotoxic drugs and boost the immune system. Although the FDA has approved various immunotherapeutic agents for treating many cancers, only a few are in clinical settings or undergoing clinical trials for the treatment of breast cancer [18].

### Immune checkpoint inhibitors

The interaction of PD1 expressed in T cells with PD-L1 on cancer cells suppresses the proliferation and survival of T cells, which ultimately leads to immunosuppression. Pembrolizumab and nivolumab are ICIs that target PD1 to prevent PD1/PD-L1 interaction [19]. In contrast, atezolizumab and durvalumab act against PD-L1 to inhibit its interaction with PD1 [20, 21]. The FDA recently approved pembrolizumab for combinatorial application with chemotherapy to treat recurrent, unresectable and metastatic TNBCs [22]. FDA has approved atezolizumab and nab-paclitaxel combination therapy for treating locally advanced or metastatic TNBCs with PD-L1-positive tumors [23]. In contrast, atezolizumab was approved earlier for the treatment of TNBC along with paclitaxel in breast cancer but later used for other cancers but not for breast [23]. In the phase I clinical trial, another ICI, avelumab, which targets PD-L1, yielded an overall response rate of 3.0%, whereas in the case of TNBC patients, the overall response rate is 5.2% [24]. CTLA4, or CD152, is another immune checkpoint constitutively expressed on Treg and activated effector T cells [25, 26]. During an immune response, particularly in the priming phase of T cell activation, CTLA4 is highly upregulated. It induces negative feedback through the binding of CD80/CD86 to prevent CD28 co-stimulation and reduce T cell activation by competitive inhibition [27, 28]. Ipilimumab and tremelimumab, two anti-CTLA4 humanized monoclonal antibodies, attenuate negative signals on T cell co-stimulation [29, 30]. Ipilimumab, in combination with nivolumab and paclitaxel, is used to treat the resistance in the early stages of TNBC [31]. Tremelimumab and durvalumab are under clinical trial to treat metastatic TNBC, as the former alone did not exhibit promising results [32]. Although TNBC has excellent response rates to ICIs as compared to other sub-types of breast cancers, the efficacy as a single therapeutic agent is still poor. Moreover, these ICIs exhibit several adverse effects such as hypophysitis, colitis, thyroid dysfunction and pneumonitis etc [33, 34].

### Monoclonal antibodies

mAbs have revolutionized cancer treatment and are widely used as immunotherapeutic agents. They target specific molecules or antigens on cancer cells and modulate the immune response to fight against cancer [35]. Trastuzumab is the first FDA-approved mAb for HER2^+^ breast cancer treatment [36]. It is employed along with other chemotherapeutic drugs to manage early-stage and metastatic HER2^+^ breast cancer [37, 38]. It inhibits the HER2 pathway to cause G1 phase arrest and inflicts apoptosis and angiogenesis in breast cancer cells by inhibiting the PI3K pathway [39, 40]. Trastuzumab also stimulates innate and adaptive immune responses through NK cells, activation of CTLs, and suppression of Treg cells [41]. However, this mAb is reported to be cardiotoxic in nature [42]. Pertuzumab is another mAb-approved drug used in combination with trastuzumab as the first-line treatment for HER2^+^ as well as non-hormonal metastatic breast cancer therapy [43]. It blocks the dimerization of HER2 with HER3 and EGFR to exhibit cytotoxic effects. This combination is also recommended for early treatment as well as trastuzumab-resistant breast cancers [44]. Margetuximab is also used in combination with chemotherapeutic agents for the management of HER2^+^ metastatic breast cancer. It enhances NK cells activity due to its high affinity for CD16A and poor binding to inhibitory CD32B [45]. This drug also activates macrophages, and neutrophils to elicit immune responses [46, 47]. Leronlimab, an anti-CCR5 antibody, is currently in a phase I clinical trial for TNBC treatment [48]. There is an ongoing clinical study to establish the efficacy of trastuzumab in combination with other chemotherapeutic drugs for treating breast cancer including TNBC [49]. Bispecific antibodies are also promising for breast cancer treatment. Zanidatamab (ZW25), targeting ECD II and ECD IV domains of HER2, is currently being clinically tested for HER2^+^ metastatic breast cancer cases (NCT04224272) [50] **(**Table 1**)**. Zenocutuzumab (MCLA-128) and KN026 are also in clinical trials for the treatment of HER2^+^ metastatic breast cancer [51, 52]. However, these mAbs rarely cause severe allergic or inflammatory reactions [49].

**Table 1:** Various Clinical Trials related to multiple therapy in different subtypes of Breast Cancer

Sl. No	Type of Therapy	Breast Cancer Subtype	Clinical Trial Identifier	Phase	References
1	ZW25 (zanidatamab) plus palbociclib plus fulvestrant	HER2+/HR+	NCT04224272	II	50
2	Tucatinib plus Trastuzumab	HER2+	NCT02614794	II	56
3	Ladiratuzumab vedotin plus trastuzumab	TNBC	NCT01969643	I	61
4	Datopotamab deruxtecan plus paclitaxel, nab-paclitaxel, carboplatin, capecitabine, eribulin mesylate	TNBC	NCT05374512	III	64
5	Datopotamab deruxtecan plus ICC eribulin, capecitabine, vinorelbine, or gemcitabine	Inoperable or metastatic HR+, HER2- breast cancer	NCT05104866	III	65
6	Glembatumumab vedotin (CDX-011) plus capecitabine	Metastatic TNBC	NCT01997333	II	67
7	Sacituzumab govitecan (IMMU-132) plus Pembrolizumab	TNBC	NCT04230109	II	68
8	Sacituzumab govitecan (IMMU-132) plus Pembrolizumab	TNBC	NCT04468061	II	68
9	Multiple drug treatment (Capecitabine, atezolizumab, ipatasertib, sgn-liv1a, bevacizumab, chemotherapy (gemcitabine, carboplatin or eribulin), selicrelumab, tocilizumab, nab-paclitaxel, sacituzumab govitecan, abemaciclib, fulvestrant, ribociclib, inavolisib, trastuzumab deruxtecan)	Metastatic	NCT03424005	Ib/II	68
10	MUC-1 peptide vaccine	TNBC	NCT00986609	I	81
11	Dendritic cells	Ductal	NCT03450044	I/II	95
12	Autologous dendritic cell vaccine	HER2-	NCT01431196	II	96
13	AdHER2/neu dendritic cell vaccine	HER2+	NCT01730118	I	97
14	HER-2/neu pulsed DC1 vaccine	HER2+	NCT02061332	I/II	98
15	HER-2/neu pulsed DC1 vaccine	HER2+	NCT00107211	I	99
16	Nab-paclitaxel	Metastatic	NCT00821964	II	101
17	Pembrolizumab plus Flt3L	Metastatic	NCT03789097	I/II	102
18	huMNC2-CAR44 CAR T cells	Metastatic	NCT04020575	I	103
19	cMet RNA CAR T cells	TNBC	NCT01837602	I	103
20	Anti-meso-CAR vector transduced T cells	TNBC	NCT02580747	I	104
21	CAR macrophages	HER2+	NCT04660929	I	106
22	Entinostat plus atezolizumab	TNBC	NCT02708680	I/II	254
23	Entinostat plus ipilimumab and nivolumab	HER2-, TNBC	NCT02453620	II	255
24	Imprime PGG plus pembrolizumab	TNBC	NCT02981303	II	290
25	PLX3397 plus eribulin	Metastatic	NCT01596751	I/II	291

### Antibody-drug conjugates

Antibody-drug conjugates (ADCs) have been developed to deliver a high concentration of anticancer drugs in the cells that overexpress the targeted antigen recognized by its antibody. This targeted delivery approach allows more efficient and selective delivery of the cytotoxic payload to cancer cells, thereby minimizing damage to healthy cells. While ADCs are not traditionally classified as immunotherapy, they utilize the immune system’s mechanisms for targeted delivery and enhanced efficacy [53]. Trastuzumab-emtansine (T-DM1), an ADC, is produced by conjugating trastuzumab with emtansine, a microtubule inhibitor and blocks HER2 signalling [40]. This ADC is approved by the European Medicines Agency (EMA) and the FDA for treating HER2^+^ early invasive and metastatic breast cancer patients as a third-line therapy [54, 55]. Combining tucatinib with trastuzumab and capecitabine increases OS and reduces brain metastasis in patients with HER2^+^ breast cancer (NCT02614794) [56]. Trastuzumab-deruxtecan (T-DXd) is another EMA and FDA-approved ADC to treat metastatic HER2^+^ and HER2-low breast cancer as a second-line therapy when surgical removal is not recommended [57]. Deruxtecan, present in this ADC, is a Topo I inhibitor, causing inhibition of DNA replication, cell cycle arrest, and apoptosis [56]. However, T-DXd frequently exhibits several adverse effects including interstitial lung disease or pneumonitis. Depending on the severity of this adverse effect, the treatment may need to be discontinued. Proper optimization of the treatment and the adverse effect management are required for maximal benefit [58, 59]. Although most ADCs, such as trastuzumab-duocarmazine, MM-302, and RC48-ADC, are based on targeting HER2, researchers are also exploring other ADCs like ladiratuzumab-vedotin and cofetuzumab-pelidotin by selecting TNBC-expressing LIV1 and PI3K as molecular targets, respectively [26]. Ladiratuzumab-vedotin (SGN-LIV1A) is another ADC, consisting of an anti-LIV-1 monoclonal antibody linked to monomethyl auristatin E (MMAE), which is a potent microtubule-disrupting agent. LIV-1 is a membrane-type metalloprotease overexpressed in most TNBCs [60]. Clinical trials are conducted in patients with LIV1 positive, unresectable, locally advanced, or metastatic breast cancers with this ADC (NCT01969643) [61]. In this trial, SGN-LIV1A is tested in TNBC patients in one arm and the HER2^+^ patients in another arm. After completion of this trial, an overall response rate (ORR) was found to be 32% and a progression-free survival (PFS) of 11.3 weeks in patients with metastatic TNBC [61]. Sacituzumab-govitecan, also known as IMMU-132, is an antibody-ADC targeting TROP2, an antigen often overexpressed in TNBC. The antibody component of sacituzumab-govitecan binds to TROP2 on the surface of cancer cells, allowing the targeted delivery of SN-38 (Topo I inhibitor) to the tumor cells [62]. This ADC has been shown to improve the ORR and PFS of metastatic TNBC patients [63]. Datopotamab deruxtecan, another anti-TROP2 mAb, is being investigated in clinical trial for unresectable or metastatic TNBC as well as HER2^+^/ HER2^−^ breast cancer cases (NCT05374512; NCT05104866) [64, 65]. It has exhibited better therapeutic efficacy with lesser adverse effects compared to sacituzumab-govitecan [64, 65]. Moreover, glycoprotein-NMB (gpNMB), significantly expressed in 40% TNBC, was explored to develop glembatumumab vedotin (CDX-011) for MMAE delivery and reported to achieve better ORR [66]. However, this ADC was less effective than capecitabine in the METRIC phase II trial (NCT01997333) [67]. Ongoing clinical trials, such as SGN-LIV1A and IMMU-132, are designed to further evaluate the potential benefits of these ADCs in treating TNBC (NCT04230109, NCT04468061, NCT03424005) [68]. Although all of these ADCs are found to be well-tolerated, these may cause cardiotoxicity, hematologic disorders, gastrointestinal problems, hepatoxicity and oral mucositis which require proper monitoring and therapeutic attention [68].

### Vaccines

HER2 has been used as a target to develop breast cancer vaccines. Due to its large molecular weight, vaccines have been generated by targeting HER2 based on one or more HER2-derived peptides. E75 or nelipepimut-S is a peptide-based vaccine that targets HLA-A2-restricted nonapeptide derived from the extracellular domain of HER2 protein [69]. GP2 is another peptide vaccine that targets HLA-A2-restricted nonapeptide based on the transmembrane domain of the HER2 protein [70]. Moreover, the AE37 vaccine is a 12-mer peptide that targets the intracellular domain of modified HER2 [71]. Four amino acids containing peptides have been added to the intracellular domain of the HER2 protein to enhance immunogenicity. HLA-A2-restricted peptides (E75 and GP2) are the epitope of MHC class I molecules and primarily activate CD8^+^ cytotoxic T cells [72]. In contrast, the AE37 peptide is presented by MHC class II molecules and primarily stimulates CD4^+^ T cell activation to elicit an immune response [73]. These vaccines are reported to be effective against low HER2-expressing breast cancer and TNBC patients [74, 75]. Researchers are actively exploring the development of vaccines targeting non-HER2 tumor-associated antigens (TAAs). Cancer-testis antigens (CTAs) are often found to be overexpressed in cancer. NY-ESO-1 is an important CTA selected to generate breast cancer vaccine [76, 77]. Other CTAs chosen for vaccine development are Wilms tumor protein 1 (WT1), the melanoma-associated antigen-12 (MAGE-12), the folate receptor alpha (FRα), T-box transcription factor brachyury and the tumor suppressor transcription factor p53 [78–80]. MUC1 vaccines have been generated based on mucin1 TAA and are being evaluated in clinical trials for TNBC (NCT00986609) [81] **(**Table 1**).** Other vaccines developed based on TAA for TNBC patients are PVX-410 (peptide vaccine) and STEMVAC (DNA vaccine) [82]. Moreover, tumor-associated carbohydrate (TAC) antigens are also used for developing vaccines. P10s-PADRE is a TAC vaccine currently in a clinical trial for TNBC patients [83]. Recently, an α-lactalbumin-targeted vaccine is also in a phase I clinical trial [84]. However, vaccines are not equally active in all patients due to spatial mutational heterogeneity within individual tumors. Therefore, personalized neoantigen vaccines are generated and tested in clinical trials against TNBC [85, 86].

Recently, DC vaccines are being developed and investigated for different cancer therapies including breast cancer. Two types of DC vaccines: DC polypeptide vaccine and DC gene vaccine, are primarily studied for breast cancer immunotherapy. One DC vaccine containing MUC1 antigen along with two adjuvants has shown to stimulate cytokine release and CD4^+^ and CD8^+^ T cell-mediated immune response in TNBC mouse model [87]. However, MUC1-based vaccines are not found to be clinically effective in early breast cancer [88]. DC vaccine loaded with P32 peptide exerts immune response using in vivo breast cancer model [89]. Another DC vaccine with MHC-II binding HER3 peptide exhibits anti-HER3 CD4^+^ Th1 immune response to inhibit tumor growth in HER3 overexpressing in vivo breast cancer murine model [90]. Oxidized cell lysate-loaded spherical nucleic acids (SNAs) act as potent immunotherapeutic agent for TNBC [91]. Exosome-loaded DC vaccines are also being investigated using in vivo breast cancer mouse and ex vivo organoid models [92]. DC vaccines developed using CD133 mRNA and MUC1 mRNA along with CTLA4 blockade have shown enhanced immune response and inhibition of tumor growth using in vivo murine TNBC models [93, 94]. Several clinical trials are in progress to evaluate the therapeutic efficacy of DC vaccines in breast cancer (NCT03450044, NCT01431196, NCT01730118, NCT02061332, NCT001070211) [95–99].

### Novel immunotherapy-based approaches

Recently, several novel immunotherapeutic strategies have been explored for solid cancers. Activation and expansion of DCs are promising immunotherapeutic approaches. TLR agonists, the potent activators of DCs have exhibited to stimulate immune response for better treatment in breast cancer [100]. Clinical trial is underway to investigate the efficacy of TLR agonist in breast cancer (NCT00821964) [101]. Moreover, these agonists are being clinically tested along with Flt3L, a stimulator of DC, for the treatment of advanced cases (NCT03789097) [102].

CAR-T, CAR-M, CAR-NK, and bispecific antibodies are reported to be very effective for breast cancer. Several CAR-T therapies are in clinical trials to investigate their safety and efficacy. The efficacy of MUC1-specific-CAR-T cells in TNBC has been established. Moreover, its safety is also being evaluated (NCT04020575) [103] **(**Table 1**)**. However, c-Met-CAR-T cells are well tolerated in TNBC patients (NCT01837602) [103]. Mesothelin-targeted CAR-T cells are in phase I study in TNBC (NCT02580747) [104] **(**Table 1**)**. CAR-M therapy, such as CAR-147, has shown reduced ECM deposition and enhanced T cell infiltration using HER2^+^in vivo breast cancer models [105]. CT-0508, an anti-HER2 CAR-M is in phase I trial for refractory HER2^+^ breast cancer patients with a parallel assignment intervention model (NCT04660929) [106] **(**Table 1**)**. CD44v6-specific CAR-NK cells have been found to be effective in a mammosphere model of TNBC [107]. However, engineered immune cell application also involves several limitations. Unavailability of suitable target, low antigen density, loss of antitumor activity due to exhaustion and poor infiltration possess challenges in CAR-T therapy [108]. Moreover, this therapy exhibits several adverse effects due to targeting antigens present in the normal tissues [109]. Common adverse effects such as neurotoxicity, thrombocytopenia, cytokine release syndromes may be life threatening [110]. C-reactive protein along with various inflammatory cytokines are also elevated [111]. CAR-NK and CAR-M also exhibit several issues like limited proliferation capacity, insufficient infiltration, limited availibility, etc [108]. 

## Tumor microenvironment-mediated immune resistance: role of immune cells

### CAF-TAM crosstalk-mediated immune resistance

#### CAFs and immune microenvironment regulation

**Fig. 1:**
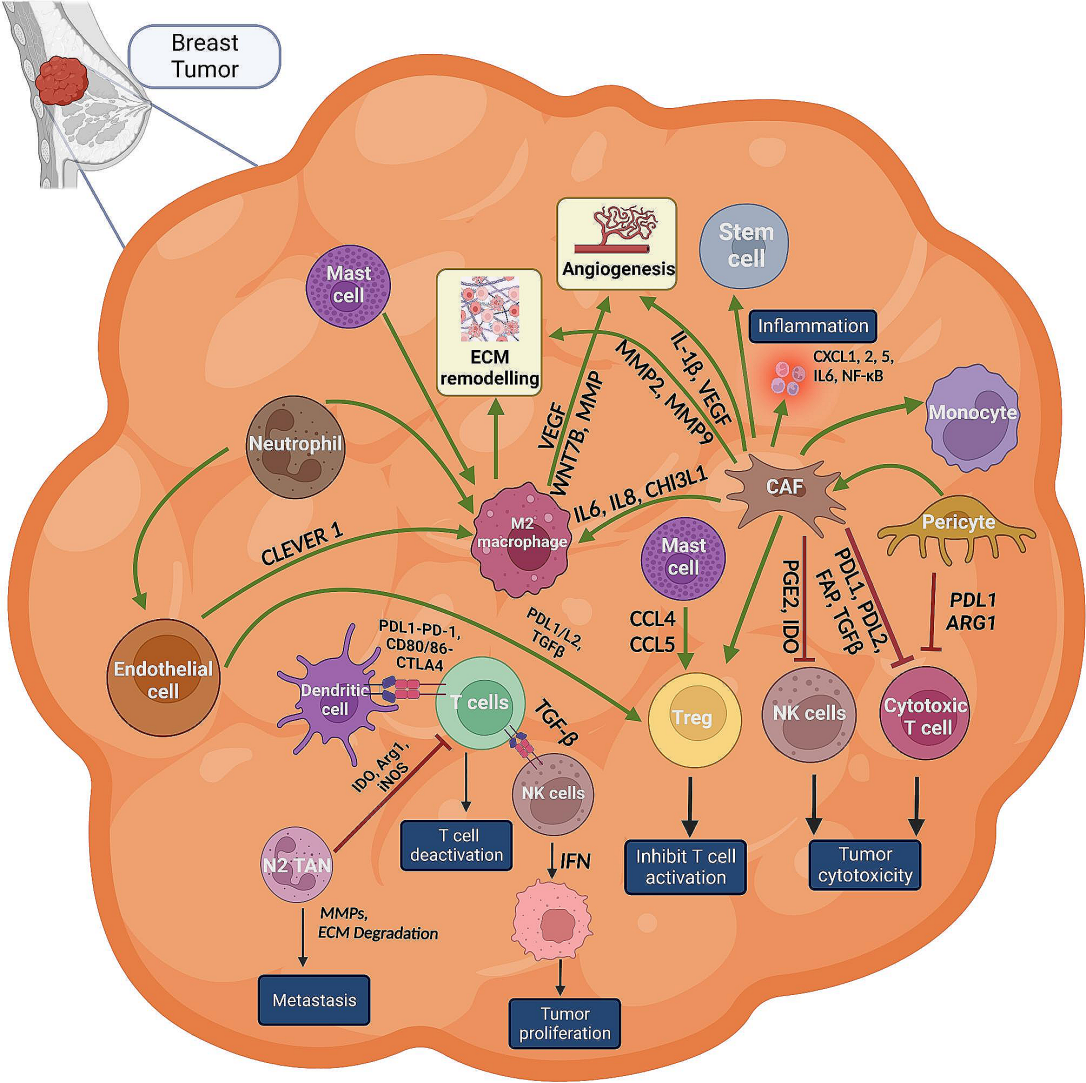
Crosstalk between different cell types in the tumor microenvironment. TAMs, CAFs, NK cells, T cells, lymphocytes, and other cells present in the tumor microenvironment modulate each other by secreting different cytokines and chemokines. This crosstalk promotes ECM remodeling and angiogenesis and causes immune suppression in the breast cancer microenvironment

CAFs, primarily generated by the trans-differentiation of resident fibroblasts, have emerged as critical therapeutic targets [10, 112, 113]. Although recent studies have identified a subset of CAFs with tumor-restricting function, modulating CAFs in combination with immunotherapy improved outcomes in different preclinical models [114–117]. Different subsets of CAFs have been identified based on the expressions of various biomarkers. Characterization and exploring the immunomodulatory role of CAF populations will be beneficial in dealing with immune resistance [118, 119] **(**Fig. 1**)**. Single-cell RNA sequencing (scRNA seq) of TNBC revealed the presence of two CAF subpopulations. One state is related to features of myofibroblasts, and the other is characterized by high expression of growth factors and immunomodulatory molecules [120]. This study has indicated the involvement of a diverse array of immunoregulatory molecules in the stromal-immune crosstalk in breast cancer. Exploring gene signatures from inflammatory CAFs (iCAFs) and differentiated‐perivascular cells revealed a strong association with cytotoxic T cell dysfunction and exclusion [120]. Costa et al. have identified four different subsets of CAFs (CAF-S1 to CAF-S4) with distinct properties and levels of activation in TNBC patients’ specimens by simultaneous analysis of six fibroblast markers (FAP, integrin β1, α-SMA, FSP1, PDGFRβ, and CAV1) [121]. Among these subpopulations, CAF-S1 exhibits the most prominent immunosuppression action. This CAF subset activates Treg cells and promotes Treg-mediated inhibition of T cell proliferation [122]. FAP^+^CAFs exhibit an immunosuppressive effect and diminish the efficacy of anti-PD-L1 therapy by secreting CXCL12 in murine pancreatic ductal adenocarcinoma (PDAC) in vivo model [115, 122]. FAP expressed by FAP^+^CAFs activates STAT3-CCL2 signaling and induces inflammatory characteristics in CAFs. This FAP^+^/CAF-S1 population boosts MDSCs recruitment, resulting in immunosuppressive TME [123, 124]. CAF-S1/iCAF subtype shapes the immunosuppressive TME by recruiting CD4^+^ CD25^+^ T cells and promoting their differentiation to Treg cells through the secretion of CXCL12 [121, 125]. Cremasco et al. have identified two distinct populations of FAP^+^ mesenchymal cells based on PDPN expression in breast cancer [126]. Myofibroblastic CAF-S1 and PDPN^+^ CAF subsets exhibit reduced IL2 activity and contribute to immunosupppression in breast cancer [127]. The FAP^+^PDPN^+^ population of CAFs is enriched at the outer edge of the tumor, in close contact with T cells, whereas the FAP^+^PDPN^−^ population of cancer-associated pericytes (CAPs) is located around the vessels. Finally, FAP^+^PDPN^+^ CAFs diminish the proliferation of T cells in a NO-dependent manner, while FAP^+^PDPN^−^ pericytes are not immunosuppressive [126].

**Fig. 2:**
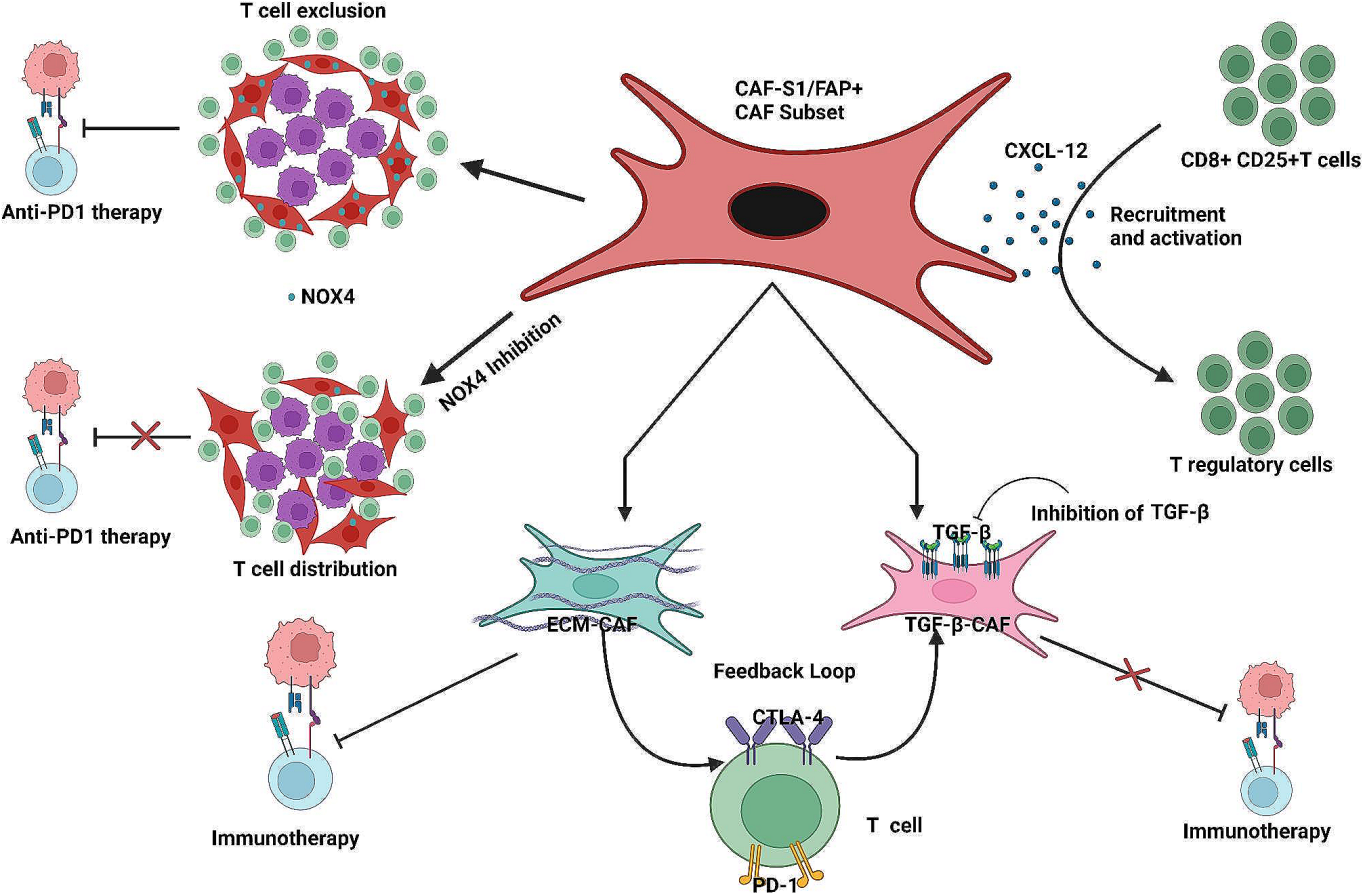
Role of different CAF subsets in immune tolerance. CAF-S1/FAP^+^ subset induces immune suppression in tumors by secreting CXCL-12, increasing the recruitment and activation of CD8^+^ CD25^+^ T cells into Tregs. CAF-S1 subtype is further classified into TGFβ-CAFs and ECM-CAFs. ECM-CAFs enhance the expression of PD1 and CTLA4 in T cells, increasing the TGFβ-CAFs. This feedback loop induces resistance to immunotherapy. CAF subsets also exclude T cells from entering the cancer cell region by providing a nest around cancer cells, leading to immunotherapy resistance. However, NOX4 inhibition leads to the distribution of T cells into cancer cell region, thereby improving the efficacy of immunotherapy in cancer

TGFβ-regulated CAFs can also contribute to immunosuppression by synthesizing ECM proteins in different types of cancers [128]. The CAFs-synthesized ECM may impact CD8^+^ T cell recruitment, thereby modulating the immunosuppressive environment [7]. A recent study has reported that CD16^+^ fibroblasts induce trastuzumab resistance in HER2^+^ breast cancer by causing matrix stiffness through VAV2 signaling [129]. Kieffer et al. have further identified eight CAF-S1 subclusters by analyzing CAF-S1 fibroblasts derived from breast cancer patient samples [122]. MyoCAFs from clusters 0 and 3 are characterized by ECM proteins and TGFβ signaling. The cluster 0/ECM-myoCAF enhances the expression of PD1 and CTLA4 in Treg cells, subsequently leading to increased TGFβ-myoCAF cellular content [122]. This study has highlighted a positive feedback loop between specific CAF-S1 clusters and Treg cells and discloses their role in immunotherapy resistance [122] **(**Fig. 2**)**. The TGFβ driven expression of leucine-rich repeat-containing protein 15 (LRRC15) is associated with poor response to immune check point blockade in PDAC [130]. Targeting TGFβ signaling in myoCAFs might be beneficial for overcoming resistance to immunotherapy as TGFβ signaling plays a critical role in the formation of myoCAFs and restriction of T cell recruitment [131]. Grauel et al. have unbiasedly interrogated tumor mesenchymal cells, delineating the co-existence of distinct CAF subsets in the microenvironment of murine carcinomas [131]. This study has shown the neutralization of TGFβ signaling significantly reduces the myofibroblast subset under in vivo conditions. However, it promoted the formation of a distinct fibroblast population that displays a more robust response to interferon and enhanced immunomodulatory properties [131]. These changes correlate with improved antitumor immunity and greater efficacy of anti-PD1 immunotherapy. It has been reported that a subset of CAFs displays the expression of PD-L1 in TNBC patients, suggesting their involvement in immunomodulation and immunotherapy response [132].

However, CAF-rich tumors also show resistance to immunotherapy by the exclusion of CD8^+^ T cells through the secretion of chemokines. CAF inhibits TNF- and IFN-induced T cell-mediated necrosis and promotes immunosuppression by activating NF-κB signaling through the secretion of IL-6 and IL-8 in human intrahepatic biliary epithelial cells [133]. Upregulation of Hedgehog signaling in CAF population leads to higher iCAF production leading to activation of Treg cells to cause immunosuppression [134]. Biglycan (BGN), a prognostic biomarker for predicting immunotherapy response is highly upregulated under immune-resistant conditions in CAFs- derived from TNBC patient’s specimens [135]. CAF-derived BGN regulates ECM remodeling and immune response in breast cancer by facilitating the interaction of CAFs with immune cells, inhibiting NK cells, CD8^+^ cells, and MDSCs while stimulating tumor-favorable macrophage activation [136].

#### TAMs and immune microenvironment regulation

Macrophages play a significant role in cancer immune surveillance. These cells are associated with a poor prognosis, malignant phenotype, and negative hormone receptor status in breast cancer [137]. TAMs are the most abundant population of tumor-infiltrating immune cells and represent the major component of the innate immune system in TME [138]. Macrophages are considered immunoreactive cells due to their phagocytic and cytotoxic characteristics. They undergo polarization in response to microenvironmental signals into classically activated macrophages (M1) and alternatively activated macrophages (M2) [139]. M1 subtypes are activated by the Th1 cytokines, including tumor necrosis factor (TNF) and interferon-γ (IFN-γ). M1 macrophages exhibit their antitumor property by producing pro-inflammatory cytokines such as TNF, interleukin 2 (IL-2), and reactive oxygen and nitrogen intermediates [140, 141]. The M2 subtypes are stimulated by the Th2 cytokines such as IL-4, IL-10, and IL-13 and express CD206 (mannose receptor), arginase 1 (ARG1), and scavenger receptors [140]. TAMs are similar to the M2-macrophages that secret pro-tumor cytokines, facilitating tumor progression [142–144]. Additionally, TAMs influence angiogenesis and promote cell proliferation and metastasis by suppressing the activity of CD8^+^ T cells [145, 146].

Several cancer cell-derived factors induce the polarization of M2 macrophages. These cells, in turn, cause tumor progression by enhancing tumor angiogenesis, immune suppression, invasion and metastasis, and ECM remodeling [147]. The functional diversity of TAMs is greatly appreciated in cancer invasion, migration, tumorigenesis, angiogenesis, therapy resistance, and tumor suppression [148]. Several studies have explored targeting TAMs in various therapeutic approaches, including immune therapy and anti-angiogenic therapy. Ongoing clinical trials are underway to investigate the therapeutic efficacy of macrophage repolarization, antibodies targeting CFSRs (a receptor of GMCSF), and macrophage depletion for cancer therapy [149]. However, novel technologies like single-cell omics have explored the information about the molecular and functional diversity of TAMs in various cancers. A recent review has reported seven TAM subsets based on their molecular signatures in almost all cancer types [150]. Among seven subsets, angio-TAMs are pivotal in promoting multiple aspects of tumor progression. The expressions of VEGF-A and SPP1 (OPN) act as molecular signature of this particular subset of TAMs [151]. In addition, TAM-associated angiogenic factors like VCAN, FCN1, and THBS1 are also reported as molecular signatures of breast cancer progression [152].

#### Role of TAMs in immunotherapy resistance

TAMs mainly affect the tumor-killing ability of effector T cells to facilitate cancer progression [153]. They primarily target arginine metabolism for inhibiting T cell activity. TAMs are found to induce ARG1-mediated hydrolysis of L-arginine in early-stage breast cancer patients. The L-arginine is essential for the functioning of the effector T cells [154]. Further, nitric oxide synthase (NOS), a molecular marker of M1 macrophages, metabolizes L-arginine to produce NO, inhibiting the activity of effector T cells [155]. Since TAMs exhibit reduced expression of MHC II using in vivo 4T1 breast cancer mice model, they are less efficient in activating T cells and antigen presentation [156, 157]. TAMs also secrete various cytokines to regulate the expression of immune checkpoints and their ligands, including PD1/PD-L1 [158]. Moreover, in vivo studies have revealed that the genetic deficiency of macrophage common lymphatic endothelial and vascular endothelial receptor 1 (CLEVER1) suppresses tumor progression by activating the tumor-killing ability in effector T cells [159]. These TAM populations impede infiltrating T cells while upregulating Treg cells in TNBC [160]. TAMs express different ligands for PD1 and CTLA4 to inhibit T cell activation [161]. They also secrete various immunosuppressive factors, including CCL20, CCL22, TGFβ, IL-6, and IL-10, that can directly inhibit both CD8^+^ and CD4^+^ T cell effector function as well as recruitment of Tregs into the tumor lesion [153, 162–165]. TAM-secreted IL-10 inhibits antigen-presenting DCs, thereby hindering tumor immunity [166]. TAM-secreted prostaglandins (PGs) and cyclooxygenase-2 (COX-2) also contribute to immunosuppression [153]. PGE2, the primary product of COX-2, is crucial for breast cancer progression as it binds to EP1-EP4 prostanoid receptors on various immune cells [167]. COX-2 inhibitors, including aspirin, can decrease the production of PGE2, which is associated with a lower risk of breast cancer progression as COX-2 is constitutively expressed at high levels in breast cancer cells [168]. Both immune and cancer cells in the TME release PGE2, which stimulates bone marrow progenitors to differentiate into MDSCs and DCs and facilitates their recruitment and activation [169]. Moreover, PGE2 induces the M2 polarisation of macrophages and their production of PD-L1. Therefore, blocking PD-L1 by anti-PD1/PD-L1 immunotherapy impairs T cell-mediated immune response against cancer [170]. However, TAMs also stimulate IL-6 by modulating PD1 signaling in response to anti-PD1/PD-L1 treatment, resulting in an immunosuppressive environment in tumors [171]. Moreover, TAMs express Fc receptors that inhibit the binding of anti-PD1 antibodies to T cells, thereby preventing the suppression of PD1/PD-L1 signaling, leading to resistance to anti-PD1 therapy using in vivo tumor models [172]. Additionally, TAMs are found to inhibit NK cell-mediated anti-tumor activity, causing immunosuppression using in vivo murine breast cancer model [153, 173].

#### Interplay between TAM and CAF in remodelling the TIME

**Fig. 3:**
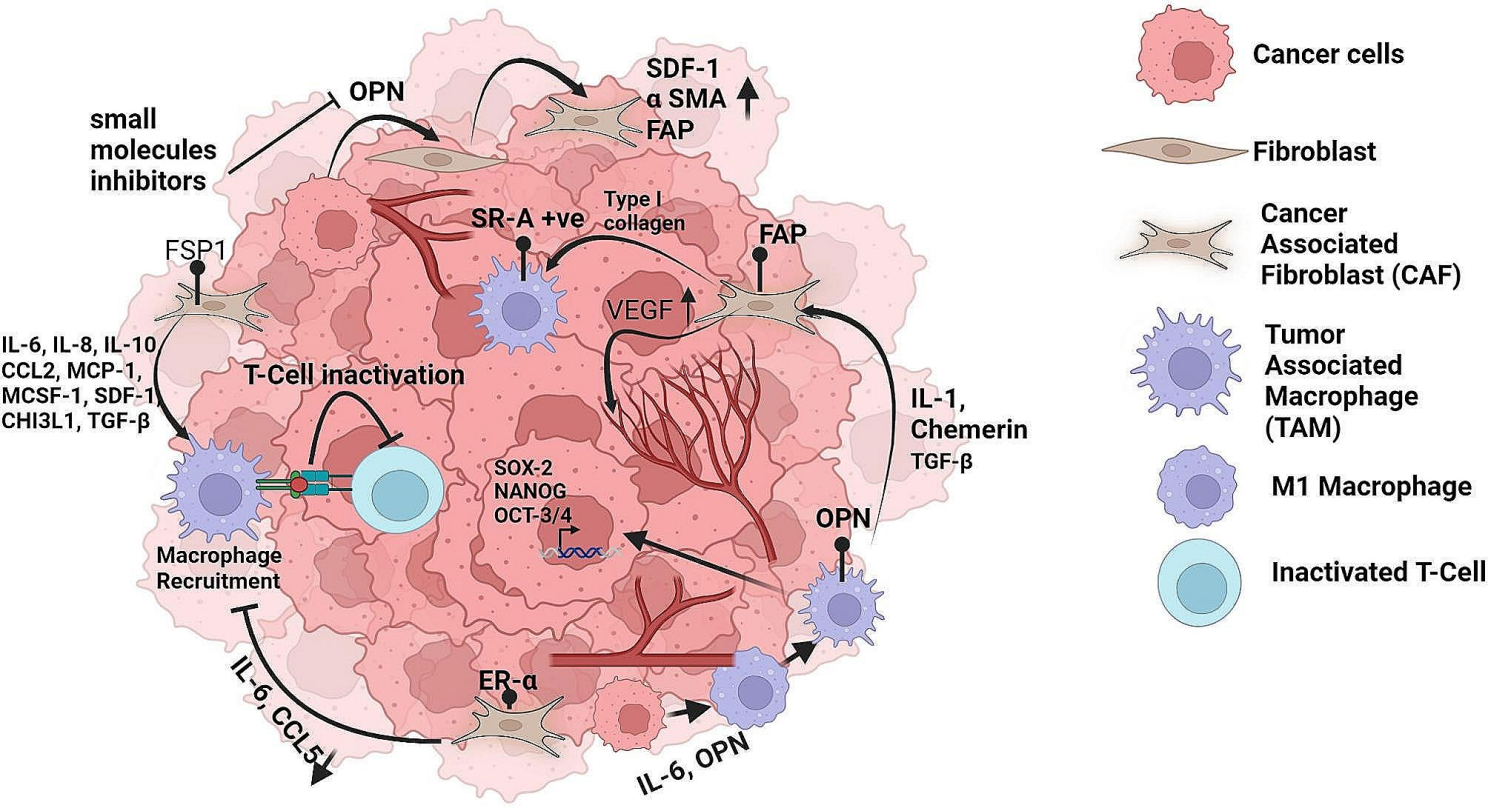
Crosstalk between CAF and TAM in the tumor immune microenvironment. CAFs trigger macrophage recruitment and differentiation through various secretory factors and regulatory networks, inducing the pro-tumorigenic capabilities of TAMs. TAMs can also induce CAF generation and activation. The interaction of CAF and TAM causes immunosuppression to promote cancer growth via a feedback loop

Tumor cells interact with stromal cells by secreting an array of cytokines, chemokines, and other tumor-promoting factors in the TME. The tumor-stromal cell interaction induces non-cancerous cells to acquire new tumor-promoting phenotypes, increasing tumor progression, multidrug resistance, distant metastasis, and immune suppression [3]. Studies using patient specimens have also shown a positive and reciprocal feedback responses among stromal cells. As discussed in the earlier section, CAFs are one of the most critical stromal cells in the TME, which is known to participate in various stages of tumor development through multiple mechanisms **(**Fig. 1**)**. Among all immune cells, macrophages play a vital role in the TIME and are known to enhance several hallmarks of cancer by infiltrating into tumors [174]. Macrophages display a wide range of plasticity and various functional activities in TIME. TAMs are the most prominent immune cells near CAF-populated areas, suggesting strong interactions between these two cell types [175]. Several studies in spheroid/ in vivo models have reported that macrophage recruitment and differentiation are triggered by CAFs via several secretory factors and regulatory networks, thereby imparting pro-tumorigenic capabilities in TAMs [176–178]. For instance, in melanoma, CAF-secreted cytokines such as IL-10, IL-8, CCL2, and TGFβ stimulate macrophage recruitment and polarisation into the M2 phenotype with tumor-promoting functions [7, 179]. Similarly, CAFs trigger monocyte recruitment and provoke differentiation of monocytes to M2 macrophages by secreting SDF-1 (CXCL12), monocyte chemotactic protein 1 (MCP1), and CHI3L1 in breast cancer [180]. The CAF-induced TAMs exhibit an elevated expression of immune checkpoints such as PD1 and cause immunosuppression by reducing T cell activation and proliferation [181]. In breast cancer, the recruitment of monocytes to the tumor is triggered by the CAF-driven CXCL12/CXCR4 axis, which also supports the acquisition of an immunosuppressive lipid-associated macrophage (LAM) phenotype [182]. MSCs acquire CAF-like phenotype through macrophage-activated signaling, inducing TME remodeling and promoting oncogenic transformation [183]. The interaction of CAFs and TAMs can enhance EMT by IL-6 and SDF-1, leading to activation of CAFs [184]. TAMs can also differentiate MSCs into CAFs through various signaling cascades [185]. Single-cell and spatial transcriptomic analyses revealed that IL-1, chemerin, and TGFβ interact with FAP^+^ fibroblasts and SPP1^+^ macrophages, allowing immune escape and restricting T-cell invasion [186]. FAP^+^ CAFs induce scavenger receptor A (SR-A)^+^ TAM adhesion via cleaving type I collagen [187]. TAMs with the M2 phenotype also control the activation and generation of CAFs [188]. In addition to their stimulatory action on TAMs, CAFs may hinder specific functions of TAMs. ERα signaling in CAFs has been shown to decrease the expression of specific cytokines and chemokines, such as CCL5 (also known as RANTES) and IL-6, that disrupt macrophage infiltration and cancer cell invasion [189]. Furthermore, M-CSF-1, IL-6, and CCL2 play a vital role in the recruitment of monocytes and the elevation of the M2/M1 macrophage ratio [190]. Additionally, co-culture of cancer cells with CAF-like BM-MSCs does not have an invasive ability but supports the proliferation of cancer cells, whereas cancer cells co-cultured with TAM-like macrophages had the opposite effect [191]. Active CAFs produced by macrophage-induced signaling boost TAM activity and create a positive feedback loop to support cancer growth and inhibit the immune response in the TME [192]. Although several reports showed the role of CAFs on TAM regulation, further studies are needed to understand the impact of macrophages on the regulation of CAF phenotypes. The CAF-TAM interaction in shaping TIME in breast cancer is elucidated in Fig. 3.

### MDSC-mediated immune tolerance

MDSCs are critical immunosuppressive components in the TME. An increased monocytic MDSC population is clinically correlated with more aggressive metastatic breast cancer [193]. These cells are activated and differentiated into atypical T cell suppressive neutrophils. Prolonged G-CSF exposure may encourage the tumor-promoting function of these immunosuppressive neutrophils [194]. MDSCs are also involved in regulating the function of B cells. They transform B cells into Breg cells to suppress T cell-mediated immune response [195]. Both monocytic and granulocytic MDSCs deplete ARG1 and induce PD-L1 expression to recruit Treg cells in the TME [196]. These cells also activate STAT3 signaling that causes T cell inhibition in response to IL6 using in vivo 4T1 breast cancer murine models [197]. MDSCs modulate MHC I to impede antigen presentation of cytotoxic T cells, resulting in immune tolerance in breast cancer [198].

### DC-mediated immune tolerance

DC is crucial component of TIME due to its antigen presenting function, leading to T cell activation and immune response. However, maturation and activation of DCs are vital for their immunostimulatory action. Immature DCs fails to activate T lymphocytes due to their high endocytic action, causing immune tolerance [199]. Immature DCs can also increase the expressions of inhibitory receptor to impede T cell activation [200]. DCs with high PD-L1 expression may inhibit CD80, leading to T cell inactivation [200]. CCL4 is associated with DC activation and T cell-mediated anti-tumor immune response [201, 202]. In contrast, CAF-secreted TGF-β interferes in the maturation of DCs, inhibiting Treg differentiation [203]. The PGE2 secreted by tumor reduces the cytokine and their receptor expressions in both NK cells and DCs to facilitate immune suppression [204].

## Role of metabolic dysregulation and epigenetic alteration in tumor microenvironment-mediated immune tolerance

### Metabolic dysregulation and immune tolerance

Due to metabolic reprogramming, cancer cells exhibit the Warburg effect, which describes the observation that cancer cells show increased glucose consumption and preferentially rely on glycolysis for energy production, even in the presence of oxygen. They use glycolytic products to promote their growth and proliferation [205]. The glycolytic glucose utilization by tumor cells limits the glucose availability in the TME, impeding T cell infiltration and IFN-γ production as T cells highly depend on glycolysis for their differentiation and effector function [206, 207]. Moreover, tumor glycolysis stabilizes Treg cells and inhibits T cell activation by modulating the glucose/lactate ratio in TME, thereby contributing to immune tolerance [208]. In low-glucose TME, Treg cells can depend on lactate for their survival and immunosuppressive action. Therefore, lactate-rich TME induces Treg polarization, leading to immune resistance [209–211]. HIF1α is also involved in metabolic reprogramming-mediated immune resistance in breast cancer. It induces PDK1 phosphorylation to inhibit pyruvate dehydrogenase, impeding pyruvate consumption in the TCA cycle [212]. Glycolytic end products such as pyruvate and lactate stabilize HIF1α to stimulate glycolysis further [213, 214]. Carbonic anhydrase IX (CAIX) is overexpressed in TNBC and promotes tumor growth, invasion, and migration. Lactate upregulates CAIX through HIF1α stabilization [215, 216]. High lactate and lactate dehydrogenase A (LDHA) expression levels stimulate HIF1α-mediated neovascularization and metastasis [217]. Therefore, lactate-induced HIF1α accumulation in TME contributes to immune tolerance. Pyrimidine metabolism, considered one of the hallmarks of cancer, is reported to be associated with immunotherapy response. Inhibition of pyrimidine synthesis results in lower CTLA4^+^ T cells in the TME [218]. Earlier studies have established the link between pyrimidine metabolism-related genes and immunotherapy efficacy [219].

**Fig. 4:**
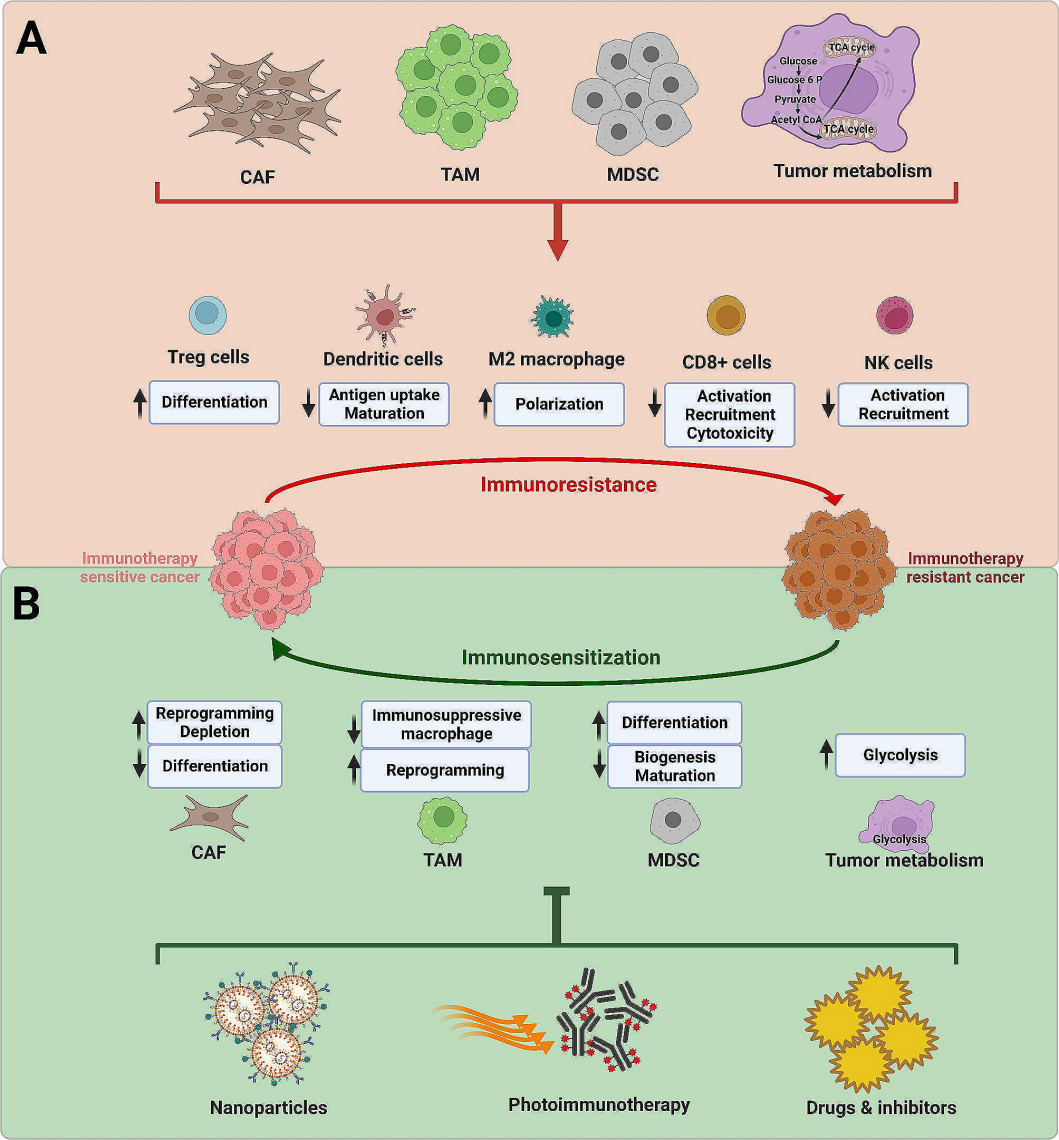
Tumor microenvironment-mediated immune tolerance in breast cancer. **(A)** Tumor microenvironment-mediated immunotherapy resistance mechanisms. Different cell types of the tumor microenvironment, such as CAF, TAM, and MDSC, contribute to immune tolerance by inducing differentiation of Treg cells, antigen uptake and maturation of dendritic cells, and M2 polarization. These cells also suppress CD8^+^ and NK cell activation and recruitment. Tumor metabolism, particularly glycolysis and TCA cycle, are also associated with immune tolerance in breast cancer. **(B)** Therapeutic strategies to overcome immunotherapy resistance. Nanoparticles, photoimmunotherapy, and several drugs and inhibitors may target CAF, TAM, MDSC, and tumor metabolism for sensitizing breast cancer to immunotherapy

Other immune tolerance mechanisms are also associated with immunotherapy failure **(**Fig. 4A**).** For example, high plasma IL-6 levels impair CD8^+^ CTL function through STAT3-mediated basic leucine zipper ATF-like transcription factor (BATF). This cytokine impedes CTL effector differentiation and gene expression, including IFNγ and perforin expression, leading to anti-PD-L1 therapy resistance in preclinical tumor model [220]. Placenta-specific 8 (PLAC8) protein also plays a pivotal role in modulating the immune response, cancer growth, and progression in TNBC [221–224]. The role of PLAC8 protein has been established using both breast cancer cell lines and patients’ specimens. This protein is overexpressed in TNBC, and its stability is regulated by ubiquitin-fold modifier 1 (UFM1), a ubiquitination modifier [225, 226]. PLAC8 has been reported to modify PD-L1 expression in TNBC via its ubiquitination, leading to immune tolerance [227, 228]. Moreover, cuproptosis, non-apoptotic programmed cell death due to high intracellular copper accumulation, is involved in immune tolerance [229]. Cuproptosis-related genes (CRGs) regulate TME and immune cell infiltration, leading to tolerance of immunotherapy. RAD23B is a vital CRG that affects immunotherapy efficacy of ICIs in breast cancer [230]. The tumors also exhibit endogenous immune response-mediated acquired immune resistance (AIR). Endogenous immune response-mediated AIR in tumors hinders the antitumor immune response and impacts overall survival rates by CD8^+^ cell-induced expression of CD163 and/or FoxP3 [231]. These data suggested a direct correlation between densities of CD8^+^ cells with CD163^+^ and FoxP3^+^ cells in the breast cancer patients’ specimens.

### Tumor mutation burden and immune tolerance

Immune suppression within the tumor niche majorly contributes to the failure of immunotherapy and its resistance. Differential expression of neo-antigens presented by APC due to mutations provokes poor T-lymphocyte recognition and activation, which results from tumor mutation burden (TMB) [232]. The T cells must be able to distinguish between tumor and normal cells after being reactivated. It is easier to identify cancer cells if their surface displays immunogenic neo-antigens. Since neo-antigens result from genomic alteration in cancer, with increases in TMB, there is an increase in immunogenicity, allowing T cells to recognize them and eradicate cancer cells [232]. However, the nature and type of mutations in TMB as a predictor in response to PD1/PD-L1 immune therapy failure in melanoma can be partially explained by the different turnover rates between genomic events and the last stages of MHC presentation [233]. In addition, it is critical to emphasize that TMB has significant limitations as a predictive biomarker, particularly when used alone, from the perspective of immune therapy response [234]. First, T cells only recognize a limited percentage of non-synonymous mutations as neo-antigens. Second, the distinct tumor molecular fingerprints and the clonality associated with these neo-antigens enhance the capacity to produce a successful anticancer therapy response [235]. Finally, the ability of T cells to penetrate the tumor site, the balance of TIME components and the equilibrium between suppressive and activating cytokines within the TIME, modulation of metabolic pathways in immune and cancer cells, and the type of checkpoint presentation by tumor influence the T cell-mediated tumor elimination [236]. Thus, while TMB is correlated with improved outcomes following ICI administration, TMB must be considered in combination with several other parameters to optimize ICI response due to the intricacy of the immune response.

### Epigenetic modification and immune tolerance

Changes in epigenetic alterations can impact the growth and development of healthy cells, potentially resulting in oncogenic transformation. Additionally, they are shown to affect immune cells’ aberrant function, normal stimulation, and activation in the TIME. These alterations modulate the activation of various signaling pathways in immune cells, affecting tumor growth [237]. Histone modification enzymes including DNA methyltransferases (DNMTs), DNA demethylases, histone methyltransferases (HMTs), histone demethylases (HDMs), histone deacetylases (HDACs), histone acetyltransferases (HATs) contribute to carcinogenesis [238]. In addition, chromatin remodeling, RNA modification, and noncoding RNAs regulate various biological processes crucial to cancer development. Zeste homology 2 (EZH2), the catalytic subunit of PRC2 provokes the release of LOXL4 in tumor cells to modulate the activation of macrophage into TAMs via miR-29b/miR-30d-LOXL4 axis in TNBC [239]. DNMT1 and EZH2 are linked with low tumor-infiltrating CD8^+^ T cells and poor patient outcomes [240]. EZH2 has also been demonstrated to play a key role in the development of Treg cells, which dampen immunological responses. Furthermore, DNMT1 attenuates the tumor cell-derived Th1-type CXCL9 and CXCL10 expression, which influences effector T cell trafficking into the TME in ovarian cancer [241].

Similarly, in TNBC, another methyltransferase known as coactivator-associated arginine methyltransferase 1 (CARM1) primarily targets BAF155 by blocking the interferon pathway, which reduces the host immune response [242]. Lysine-specific demethylase 1 (LSD1), a histone demethylase involved in epigenetic EMT regulation, the acquisition of cancer stem cell markers (CSCs), and treatment resistance in breast cancer, could be a promising target for overcoming anti-PD-L1 therapy resistance. LSD1 is inversely correlated with CD8^+^ T cells in breast cancer, non-small-cell lung cancer, and melanoma [243]. By suppressing the MHC-I-producing genes, H2-D1, H2-K2 and LSD1 significantly impact the normal expression of MHC-I protein antigen in tumor cells. This enhances the exclusion of MHC-I identification by CD8^+^ T lymphocytes, which could result in immunological escape [244]. The K acetyltransferase 6 A (KAT6A) acetylation of SMAD3 controls macrophage recruitment, metastasis, and immunosuppression. Combining anti-PD-L1 therapy and KAT6A inhibitor reduces metastases and improves survival in TNBC xenograft-bearing mice [245]. TNBC is caused by hypermethylation of the DNA methyltransferase 1 (DNMT1) gene [246]. In addition, the histone demethylase, KDM5B has been shown to promote the migration, proliferation, and modulation of cellular physiology of tumor cells. Therefore, KDM5B silencing in breast cancer reprograms lipid metabolism to stimulate the migration and proliferation of breast cancer cells via activation of AMPK [247]. BRD4 inhibition has been found to induce macrophage reprogramming from the M2 to M1 phenotype and proinflammatory cytokine production, resulting in T cell activation. Similarly, this suppression was correlated with increased expression of MHC 1 genes by tumor cells and an increase in the CD8^+^ T cells/Tregs ratio [248].

### Combination of immunotherapy and epi-drugs in immunotherapy

Extensive heterogeneity has impeded the management of TNBC by causing therapy resistance. Combination drug therapy (or CDT) has gained an improved pathological clinical response (PCR), progression-free survival (PFS), and overall survival (OS) in various malignancies [249]. Epigenetic drugs or epi-drugs are shown to have promising results in various solid cancers, including breast. Goswami et al. have uncovered an additional benefit of combining immunotherapy with EZH2 inhibitors [249]. They have shown that combination treatment might boost the therapeutic efficacy of antibodies that target CTLA-4 and decline the number of immune-suppressive cells [249]. In recent days, bromodomain and extra-terminal (BET) inhibition have gained significant attraction in the treatment of breast cancer. Several BET inhibitors, such as birabresib, molibresib, and mivebresib act by modulating the interaction between the enhancer and promoter for transcriptional repression [250]. Additionally, new strategies that target chimeric chemicals (BET-PROTACs) by BET-proteolysis have been explored in TNBC with encouraging results, even in BET-resistant cancers, and they bind a ubiquitin ligase while allosterically inhibiting BET bromodomains [251]. BET inhibitor resistance in TNBC is correlated with TAM infiltration in the TME [252]. Entinostat, a HDAC inhibitor augments the anti-tumor effects of IL-15 agonist and vaccine in 4T1 TNBC mouse models [253]. In addition, clinical trials investigating this epi-drug in combination with atezolizumab showed overall response rate of 10% (NCT02708680) [254]. A recent phase Ib trial with entinostat, nivolumab along with ipilimumab in hormone receptor positive and advanced TNBC suggested the overall response rate of 25%, with 10% in hormone receptor positive and 40% in TNBC and recommended a further study of these combinations in phase II trial (NCT02453620) [255].

Furthermore, additional investigation is needed to determine whether ICI, along with different epi-drugs specifically targeting each type of immunosuppressive cells may be more beneficial for breast cancer therapy. These cutting-edge therapeutic strategies may be enhanced by developing the precisely targeted drug delivery systems for the treatment of breast cancer.

## Therapeutic strategies to overcome immunotherapy resistance

Several groups have explored developing new therapeutic strategies for immunotherapy tolerance. CMTM7, a PD-L1 regulator, is frequently deleted or downregulated in TNBC to cause therapeutic resistance. TNBC with higher CMTM7 expression is more sensitive to chemotherapy and immunotherapy [256]. This protein exhibits a positive correlation with immune cell infiltration and immune checkpoints. High CMTM7 protein level leads to a better therapeutic response of anti-PD1 or anti-PD-L1 therapy. Hence, CMTM7 can be considered a predictive biomarker for immunotherapy response, and its expressions can be modulated to deal with immune tolerance in breast cancer [256]. PSME2, overexpressed in breast cancer, is involved in the proteasomal degradation of several proteins. It positively correlates with immune response and good prognosis in HER2^+^ breast cancer patients [257]. It promotes immune cell infiltration and checkpoint functions, improving immunotherapy outcomes. This novel biomarker in breast cancer may also be modulated to overcome immune resistance [257]. Earlier reports have revealed three TAAs: CD74, IRF1, and PSME2, associated with immune cell infiltration in breast cancer. These three TAAs are found to be overexpressed, amplified, or mutated in breast cancer and directed for developing mRNA vaccines in this cancer [258]. Moreover, HSP90 inhibitors reduce PD-L1 and PD-L2 surface expressions and increase CD8^+^ T cell infiltration in the tumor [259]. NDNB1182, an HSP90β inhibitor, blocks CDK4 and stimulates the expressions of IFN-mediated genes. HSP90 inhibitor with immune checkpoint blockade is employed for treating immunotherapy-resistant murine breast cancer [260]. Another natural product-based HSP90 inhibitor, 17-AAG, is found to be effective along with trastuzumab in treating trastuzumab-refractory HER2^+^ breast cancer [261]. However, it exhibited dose-limiting hepatotoxicity and gallbladder toxicity in preclinical study [262]. TNFα is also involved in trastuzumab resistance in HER2^+^ breast cancer. It upregulates the expression of MUC4, which interacts with HER2 through its MUC4β subunit and promotes tumor metastasis [263, 264]. MUC4 shelters the trastuzumab epitope in HER2 protein, blocking trastuzumab interaction [265]. Soluble TNFα-mediated inhibition of MUC4 downregulation modulates macrophages and NK cells to reverse immunosuppressive environment and trastuzumab resistance [266]. MSA2, a stimulator of interferon genes, boosts dendritic cell maturation and its antigen-presenting ability. It also promotes macrophage activation along with the release of chemokines and cytokines [267]. Improved T cell migration with chemotaxis leads to a better innate and adaptive immune responses against breast cancer. NSA2 is used with YM101, an anti-TGFβ/PD-L1 antibody, to deal with immune resistance in non-inflamed tumors [267]. Direct Akt activation stimulates the immune system in PD1 checkpoint blockade-resistant tumors to suppress their growth. The Akt downregulates Treg cells while upregulates CD4^+^ and CD8^+^ tumor-infiltrating lymphocytes (TILs), leading to IFNγ expression and thereby inducing an anti-tumor immune response [268]. A combination of anti-PD-L1 monoclonal antibodies and PARP inhibitors are also effective in treating breast cancer patients [269]. Combinatorial treatment with albumin-paclitaxel and pembrolizumab also exhibits efficacy in TNBC cases exhibiting higher PD1 on T cells [270]. Moreover, determining the drug exposure of tumor tissue is important to understand the mechanism of immune tolerance. [^68^Ga]Ga-DK223-PET has been developed using Gallium-68-labeled peptide and investigated for monitoring tumor status with anti-PD-L1 therapy. This strategy helps to optimize immunotherapy for effective treatment [271]. A non-invasive method has been developed for analyzing the blood-based TMB and copy number profiling to predict outcomes in breast cancer patients undergoing treatment with the combination of endocrine therapy along with CDK4/6 inhibitor, a standard treatment for HR^+^/HER2^−^ metastatic breast cancer [272, 273]. Along with these approaches, there are several reports employing various therapeutic strategies to overcome immunotherapy tolerance.

### Targeting CAFs

CAFs have been shown to contribute to immunotherapy tolerance significantly. Researchers have developed various therapeutic interventions to improve immunotherapy response by targeting the CAF population in the TME. Ford et al. have assessed the potential of CAF targeting the NADPH oxidase 4 (NOX4) inhibition in several cancers [274]. NOX4 is an enzyme involved in the differentiation of myoCAFs, and it’s inhibition reverts the myoCAFs into a quiescent phenotype and promotes intra-tumoral infiltration of CD8^+^ T cells. NOX4 inhibition has shown to overcome the CD8^+^ T cell exclusion and potentiate anti-PD1 immunotherapy response using in vivo murine breast cancer model [274] **(**Fig. 2**).** GKT137831 is a NOX4/1 inhibitor reported to repress CAF-mediated immune tolerance [275, 276]. TGFβ1-induced ECM remodelling in CAFs stimulate hepatocellular cancer cell invasion [277]. Upregulation of IL2 in TGFβ^+^ and PDPN^+^ CAF populations sensitizes the trastuzumab-resistant HER2^+^ breast tumor [127, 278]. TGFβ inhibitors may also address immunotherapy resistance by targeting the CAF population [127]. The depletion of CAF is alternative strategy for improved immunotherapy efficacy. Depleting FAP^+^ CAF has been reported to boost the effectiveness of vaccines against cancer [117]. AMD3100 blocks the action of CAF-mediated CXCL12 signaling and immunosuppression by targeting CXCL12-CXCR4 interaction [115, 279]. However, targeting CAF has certain limitations due to the existence and insufficient understanding of various CAF subpopulations; therefore, precisely targeting CAF is more challenging. Selective CAF subpopulations possess anti-tumorigenic properties, leading to ineffective therapy [280]. Most CAF-targeted therapy could be combined with other immunotherapy for successful breast cancer treatment [281]. Lack of preclinical and clinical data is a limitation affecting CAF-targeted therapy [282].

### Targeting TAMs

TAMs are reported to involve in immune resistance through various mechanisms. Several groups have explored many therapeutic options along with anti-PD1/PD-L1 therapy [283, 284]. However, a detailed understanding of the heterogeneity of TAM needs to be explored further [285, 286]. Single-cell RNA seq has established the existence of both immunosuppressive and immunostimulatory TAM subpopulations at the tumor site. CD8^+^ T cell enrichment reduces memory T cells and inhibitory macrophages among various TAM populations [287]. Folate receptor 2 expressing macrophages (FOLR2^+^) is involved in the anticancer immune response. FOLR2^+^ macrophages induce higher CD8^+^ T cells and dendritic cell infiltration into the breast tumor niche [288]. In contrast, CX3CR1^+^CCR2^−/low^ TAMs induce tumor-promoting TME [286]. TREM2^+^ macrophage subpopulation contributes to immune resistance via exhausting T cells [283]. The preclinical result of emactuzumab, a CSF1R antibody, established its efficacy in targeting the CD163^+^ TAM population in breast cancer [289]. BTH1677, an agonist of Dectin receptor and pembrolizumab treatment, exhibits repolarization of M2 macrophages, and a phase II trial is under process to study the efficacy of this combination therapy in metastatic TNBC (NCT02981303) [290]. PLX3397 (Pexidarnitib), another anti-CSF1R antibody, is also being investigated along with eribulin for the treatment of brain metastatic cases of breast cancer (NCT01596751) [291]. Although several reports are available on repolarization, reprogramming, and depletion of TAM in cancer, these strategies are not sufficient enough to overcome breast cancer immune resistance.

### Targeting MDSC

A cryo-thermal treatment strategy has been developed to target metastatic tumors by activating innate and adaptive immune responses [292]. This therapy inhibits MDSCs by inducing their differentiation into antigen-presenting cells. However, to improve the in vivo efficacy of this therapy, all-trans retinoid acid (ATRA) is employed to stimulate the maturation of functional MDSCs and inhibit immunosuppressive molecules [293]. This combination treatment inhibits Th2 and Treg subpopulations while stimulating cytotoxic CD8^+^ T cells and NK cells to address MDSC-mediated immune tolerance [293]. Moreover, MDSC biogenesis may be targeted to reduce MDSC load and enhance immunotherapy response. Dihydroorotate dehydrogenase inhibitors downregulate MDSC generation and maturation, improving immunotherapy efficacy in an in vivo TNBC model [294] **(**Fig. 4B**).** Brequinar, an inhibitor of dihydroorotate dehydrogenase, has been reported to enhance the effectiveness of immune checkpoint inhibition in refractory HER2^+^ breast cancer [295]. ADAM12 is a metalloproteinase that is highly expressed in TNBC [296]. This protein is negatively correlated with the expression of MDSC genes. ADAM12 inhibition suppresses MDSCs and improves T and B cell infiltration at the tumor site using an in vivo TNBC model. Combination of anti-PD1 and anti-CTLA4 therapy has enhanced the efficacy of immunotherapy upon abrogation of ADAM12 using in vivo murine breast cancer model [297].

### Modulation of metabolism

Tumor metabolism plays a crucial role in regulating tumor immunity and immunotherapy response. The high nutrient demand of cancer cells for their rapid growth and proliferation limits the availability of metabolic nutrients for immune cells, leading to diminished immune activity [298]. Cancer cell metabolism suppresses T cell metabolism and its immune function. Tumor cells with high glycolytic activity affect the growth of T cells by depleting glucose in TME to impede T cell-mediated cytokine secretion. Targeting CTLA4 and PD-1 improve T cell metabolism and restore its immune function [28]. Amino acid metabolism is also crucial for the function of T cells [299]. TAM, especially M2 macrophages, are associated with metabolism-dependent immunosuppression. M2 macrophages reduce the glycolytic flux by inducing PD-L1 expression to avoid the competition for oxidative phosphorylation. High PD-L1 expression in TAMs leads to immunosuppression [207, 300]. CSF1R inhibitors have the potential to block the transcriptional activation of genes involved in TAM-mediated immunosuppression [146]. M1 repolarization may also effectively suppress M2-dependent metabolic modulation of TIME [158]. Lipid metabolism also significantly affects ICI therapy by hampering T cell proliferation and activation. Metabolites may also act as a signaling agent to modulate immune activity [301]. Several reports are available on metabolic reprogramming for enhanced immunotherapy response. CD28 and CTLA4 are involved in competitive regulation of metabolism by binding with common receptors, CD80 and CD86. Highly abundant CD28 exhibits a lower affinity to these receptors, while less abundant CTLA4 shows a higher affinity of it [302]. CD28 promotes glucose utilization through glycolysis in T cells, which is required for their activation [303]. CTLA4 inhibits CD28-mediated glucose metabolism in T cells through Akt inhibition, impeding T cell activation [304]. Anti-CTLA4 therapy promotes CD28 stimulation in antigen-presenting T cells and hampers glucose-dependent Treg cell stabilization [206]. Hence, CTLA4 inhibitors can be usefull in dealing with immune tolerance in breast cancer [305]. Tumor glycolysis can also be targeted to inhibit MDSCs infiltration-mediated immunosuppression in TME by LDHA knockdown in TNBC [306]. LDHA knockdown also destabilizes HIF1α to promote immune cell infiltration, improving immunotherapy outcomes in murine breast cancer [217, 307]. FX-11, an LDH inhibitor, is combined with anti-PD1 therapy to induce cytotoxic CD8^+^ T cells and NK cells, resulting in improved antitumor immune response in TNBC [308]. It has been reported that CAIX inhibition can boost immune checkpoint inhibitor’s efficacy in TNBC. Preclinical data have shown that SLC-0111, a CAIX inhibitor, acts synergistically with immune checkpoint inhibitors such as anti-PD1 or anti-CTLA4 to suppress tumor vascularization and metastasis in a TNBC xenograft model [309, 310].

### Photodynamic therapy and photoimmunotherapy

Photodynamic therapy (PDT), which employs a photosensitizer to generate light-induced ROS, along with immunotherapy overcome the immune tolerance [311]. PDT-induced oxidative stress leads to calreticulin-mediated necrosis of tumor cells and secretes damage-associated molecular patterns (DAMPs) to cause antigen-presenting T cell activation [312]. PDT also selectively activates macrophages in a dose-dependent manner [313]. It stimulates macrophages to secrete lysophosphatidylcholine (LPC), which forms macrophage activating factor (MAF) via T and B cell signaling to induce anticancer effects. PDT-induced phagocytosis in macrophages results in CD8^+^ T-cell activation [314]. PDT inhibits immunosuppressive TME along with its anti-tumor immune response [311]. It is used with immunomodulatory agents to prevent metastasis through improved CD8^+^ T cell stimulation [315].

Phototherapy combined with immunotherapy (photoimmunotherapy; PIT) is applied to escape the immunosuppressive TME. PIT activates the system’s immune response to achieve long-term antitumor immunity. It also targets metastasized tumors and prevents breast cancer recurrence [316]. Two-dimensional black phosphorus (BP) nanostructures have gained popularity for their PIT applications. CpG oligodeoxynucleotide encapsulating NIR and ROS-sensitive BP nanovesicles (BPNVs) stimulates cytokine release by antigen-presenting cells (APCs) [317]. It generates NIR laser irradiation-responsive ROS to release CpG at the tumor site. APCs take up the released CpG to activate the cytokine-mediated immune response against the tumor. The in vivo efficacy of this nanosystem is also established using in vivo 4T1 tumor-bearing BALB/c mice models [318].

Photothermal therapy (PTT) can also be efficiently employed for potential tumor immunotherapy. A biomimetic NIR-responsive black phosphorus quantum dots (BPQDs) formulation is developed by coating with erythrocyte membrane to achieve better tumor accumulation and prolonged circulation [319]. It induces PTT for immune system activation in breast cancer to target metastatic and residual tumors. NIR irradiation leads to dendritic cell recruitment and elicits CD8^+^ T cell response at the tumor site. A combination of PD1 therapy and BPQDs exhibits more potent activity against primary and secondary cancers [319]. In addition, tumor-specific PTT is developed using cancer cell membrane coating for BPQDs [320]. PD-L1 combination with BPQDs improves dendritic cell maturation, and T cell-mediated anticancer immunity leads to better tumor cell recognition and tumor-specific lethal efficiency. This immunotherapy combination exhibits an immunological memory effect, resulting in more efficient action for recurrence and metastatic TNBC [320]. Another BPNP is fabricated using PEGylated hyaluronic acid (HABPs) and applied with PTT, PDT, and PIT. This formulation downregulates CD206 expression and upregulates CD86, leading to the repolarisation of TAMs into M1 macrophages. In vitro and in vivo studies demonstrated that combining PDT, PTT, and HABPs immunotherapy causes immunogenic cell death. This combination therapy secretes DAMPs for robust anticancer immunity through improved dendritic cell maturation and effector cell stimulation [321]. All these potential therapeutic strategies to overcome immune tolerance in breast cancer have been depicted in Fig. 4B.

### Regulation of tumor immune genes and immunotherapy response

Recent advances using i*n vivo* CRISPR screens have identified various genes that make cancer cells evade anti-tumor immunity or regulate response to ICI therapy. Deletion of these tumor immune genes improves the immunotherapy outcome. In vivo CRISPR knockout screening using a syngeneic TNBC mouse model has revealed the role of E3 ubiquitin ligase, Cop1, in regulating immunotherapy response. The first screen utilized a customized lentiviral guide RNA library called MuSCK, targeting 4500 genes involved in cancer progression and immune evasion. The library was transduced into 4T1 cells and then injected into mice. A subsequent secondary screening with the MuSCK 2.0 library was performed to validate 79 hits. Cop1 deletion also improves the anti-tumor immune response by inhibiting chemokine secretion and macrophage infiltration in the TME [322]. Another in vivo CRISPR screen employed a guide RNA library, DrIM, targeting 2796 human disease-associated immune genes transduced into 4T1-Cas9 cells. Validation in immunocompetent and immunodeficient mice identified Ido1 and Lgals2 as immunotherapy targets in TNBC. These genes also promote TAMs and M2 polarization [323]. Dong et al. conducted an unbiased genome-wide CRISPR screening using a single guide RNA library called mouse knockout (MKO) library [324]. E0771 murine TNBC cells expressing the ovalbumin tumor antigen were employed, and Cas9-expressing CD8^+^ T cells, transduced with the MKO library, were injected into mice with E0771-ovalbumin transplants. The screening has identified Dhx37 as a regulator of T cell function in the TME. The study established that Dhx37 dampens the CD8^+^ T cell activity by physically interacting with the NF-κB pathway in TNBC [324]. A pooled in vivo CRISPR screen approach has identified defective IFNγ signaling responsible for immunotherapy resistance. The protein tyrosine phosphatase PTPN2 deletion improves IFNγ-mediated antigen presentation, leading to better immunotherapy response [325].

### Combination therapy and nanoparticle-mediated immunotherapy

Immunostimulatory and immunomodulatory molecules must be precisely and efficiently delivered to the right target immune cells to employ cutting-edge therapeutic strategies in reshaping TIME without off-target effects. Moreover, nanocarrier-mediated combinatorial approaches are very promising to achieve better immunotherapy response. Kim et al. have shown the efficacy of SGT-53 to potentiate the action of anti-PD1 therapy using in vivo 4T1 breast cancer model [326]. SGT-53, a nanocarrier containing a plasmid encoding p53 gene, stimulates immune response and sensitize the resistant tumor to anti-PD1 antibody. Moreover, this combinatorial treatment also limits the immune-related adverse effect [326]. Combination of chemotherapy and immunotherapy is also investigated to improve the treatment outcome. Phase III trial was conducted with the atezolizumab and nanoparticle albumin-bound paclitaxel (nab-paclitaxel) for the treatment of metastatic TNBC. The results have shown the improved efficacy of atezolizumab in TNBC particularly in PD-L1^+^ tumors [327]. pH responsive micellar nanosystem has been developed for co-delivery of anti-PD-L1 siRNA and photosensitizer. This nanocarrier elicits PDT-induced antitumor immune response and overcomes the immune tolerance in melanoma [328]. Kang et al. have investigated the potential of chemoimmunotherapy using nanoparticulate system [329]. Paclitaxel and imiquimod have been co-assembled for improved immunotherapy response as well as antitumor efficacy [329]. Nanomaterials have also been shown to possess an intrinsic ability to influence immune cells directly, including macrophage polarisation, thus becoming an appealing strategy to reprogram TAMs [330]. Various metallic nanoparticles are reported to activate immune response. Iron oxide nanoparticles have shown to encourage immunosuppressive M2 TAMs to repolarize into the pro-inflammatory M1 phenotype using in vivo murine breast cancer model [330]. In addition, a study has shown that gold and silver nanoparticles can provoke an immune response in TAMs. Gold and silver nanoparticles contribute to TAM reprogramming into M1-like phenotype through downregulating TNF-α and IL-10 and upregulating IL-12 [331]. Treg depletion or suppression of their immunosuppressive actions may potentially restore effector T cell antitumor activity, inhibiting tumor growth. Restoration of antitumor immune response within the TIME is a challenging task. Recently, a nanoparticle-based MUC1 mRNA vaccine (NP) in combination with anti-CTLA-4 has shown to induce cytotoxic T lymphocyte response, leading to enhancement of antitumor activity as compared with vaccine or anti-CTLA4 alone in 4T1 mice models [94]. Understanding the drug targets and off-targets in immune and stromal cells requires robust drug delivery systems within the TIME. Therefore, selecting suitable delivery systems, dose optimization, drug combinations, and their pharmacokinetics and pharmacodynamics in TIME will open new avenues to overcome immunotherapy resistance in breast cancer.

## Conclusion and future perspective

Immunotherapy has therapeutic potential for treating breast cancer, resistance to conventional therapies. Several lines of investigation are in progress for an in-depth understanding of the immune tolerance mechanisms to develop novel therapeutic approaches for combating immune resistance. The study of intricate biological phenomena has been revolutionized by single-cell omics tools, such as assay for transposase accessible chromatin (ATAC) sequencing and single-cell RNA, which enable detailed monitoring of the TME during immunotherapy. Incorporating single-cell CRISPR screens improves the resolution of biological event analysis, while concurrent spatial omics studies offer simultaneous spatial information and protein expression. Researchers have carried out a parallel single-cell RNA-seq and T cell receptor (TCR) profiling using breast cancer patient samples to understand the tumor microenvironmental changes following anti-PD1 therapy [287]. Single-cell analysis revealed specific immune cell subsets linked with anti-PD-L1 therapy in TNBC [332]. This study identified responders with an increased pool of CXCL13^+^ T cells by mapping alterations in immune cells after anti-PD-L1 and paclitaxel therapy using paired single-cell transcriptome, ATAC, and TCR sequencing [332]. Additionally, single-cell RNA-seq has clarified the intricate biology of T cell exhaustion in the TME [333]. Algorithms facilitating co-registration of single-cell and spatial NGS data offer unprecedented insights into the co-evolution of immune cell clones. They may aid in identifying targets for combination therapy in cancer immunotherapy regimens. Therefore, a detailed understanding of the tumor microenvironmental changes during immunotherapy through single-cell omics, multi-omics, or multiplexed in situ spatial protein profiling may aid in developing novel therapeutic strategies to overcome immune tolerance in breast cancer.

The intricate interplay between CAF and TAM is crucial in determining immune therapy efficacy with the TIME. For instance, a pan cancer analysis revealed iCAFs stimulate cancer cell proliferation, epithelial-mesenchymal transition (EMT), and the create an immunosuppressive TIME in breast cancer patients receiving anti-PD1 immunotherapy [334]. Similarly, Li et al. have characterized two CAFs clusters A and B where cluster B is abundant with immunosuppressive macrophages and resulted in poor overall survival than cluster A [335]. TAM depletion by sequential administration of TAM targeting T cells followed by cancer targeting T cells, resulting in reduction in tumor size and longer survival in mouse ovarian cancer model [336]. CAF-derived IL-6 contributes to immune therapy resistance and inhibition of the IL-6-STAT3/Akt-PD-L1 axis resensitizes to immune therapy in breast cancer patients [337]. In addition, MDSC is an essential component of the TME to be considered in immunotherapy outcomes. Tumor metabolism, gaining attention in cancer research, is also linked to immunotherapy resistance. Thus, developing strategies targeting CAF and TAM regulated core transcriptional networks and its heterogeneity could provide better immune therapy-based strategy for the treatment of breast cancer. Immune therapy resistance is also conferred by epigenetic modifications in various cells within the TIME. Particularly immune cells are regulated epigenetically in modulating immune cell function and developing immune therapy resistance. Combining epi-drugs along with various immune therapy-based approaches could provide better disease-free survival in breast cancer patients.

Nanotechnology is also being used to reprogram TME for immune stimulation. Nanoparticles can contribute to better drug uptake, improved CAF reprogramming, and better T cell infiltration, leading to more effective immunotherapy. PDT, PIT and PTT improves the immunotherapy efficacy thereby providing better therapeutic outcomes in breast cancer. Combining nanoparticles with photodynamic immunotherapy ablates TAM metabolism in TNBC [338]. Further addressing PDT related adverse effects in terms of structural and biological aspect while combining with immunotherapy could improve clinical manifestations in breast cancer treatment [339].

Moreover, non-specific immunostimulation by immunotherapy mimics autoimmune disorders and causes several immune-related adverse effects. Minor toxicity may be managed by temporary withdrawal of immunotherapy while it must be discontinued in case of severe toxicity. Different immunotherapies have their specific immune-related adverse effects. Along with immunotherapy resistance, addressing immune-related other adverse effects is also crucial for successful immunotherapy outcome [340]. All these factors should be considered to control the TIME for a better immunotherapeutic outcome.

Additionally, organ-specific differences in TME may cause different immune-resistant mechanisms and immune therapy responses [341]. Hence, we must understand the organ-specific TME to establish more precise therapy depending on the immune tolerance mechanism. Immuno-subtyping may be a successful approach to deal with immunotherapy failure. It will aid in understanding tumor heterogeneity among breast cancer patients [342]. Moreover, single-cell and spatial transcriptomics studies may detangle the complexity of TME-mediated immunomodulation. These will be beneficial to explore biomarkers for immunotherapy response prediction and targets for improved immunotherapy outcomes [343]. Advanced therapeutic strategies, including personalized immunotherapy and gene editing based therapeutics, may be helpful to modulate different TME components for enhanced immunotherapy efficacy.

In conclusion, various components of TME such as CAFs, TAMs, DCs and MDSCs are involved in TIME modulation, leading to immunotherapy resistance. In this review, we have highlighted various immunotherapy-based approaches and stromal-immune interplay such as CAF, TAM and MDSC-mediated immune tolerance in breast cancer. Moreover, alteration of tumor metabolism leads to immunotherapy failure. Furthermore, targeting these and their metabolic regulation with combination therapy could overcome immune resistance and enhance the efficacy of immunotherapy in breast cancer. This review emphasizes the therapeutic approaches to overcome breast cancer immune resistance in combination with immunotherapy such as photodynamic, photoimmunotherapy, epi-drug and nanoparticle mediated drug delivery. The comprehensive strategies emphasize that the resistance to immunotherapy needs to be further studied to develop therapeutic regimens for the successful overcome of immunotherapy resistant breast cancers.

## Data Availability

Not applicable.

## Abbreviations

CAF: Cancer-associated fibroblast
TAM: Tumor-associated macrophage
GLOBOCAN: Global cancer observatory
TME: Tumor microenvironment
DC: Dendritic cell
MC: Mast cell
ECM: Extracellular matrix
TAN: Tumor-associated neutrophil
PD1: Programmed cell death protein 1
PDL1: Programmed cell death protein ligand 1
Th: T helper type
NK: Natural killer
CTL: Cytotoxic T lymphocyte
Treg: Regulatory T cell
MDSC: Myeloid-derived suppressor cell
ICIs: Immune checkpoint inhibitors
TIME: Tumor immune microenvironment
mAB: Monoclonal antibody
ADC: Antibody-drug conjugate
EGFR: Epidermal growth factor receptor
CTLA4: Cytotoxic T-lymphocyte–associated antigen 4
FDA: Food & drug administration
T-DM1: Trastuzumab-Emtansine
TNBC: Triple-negative breast cancer
HER: Human epidermal growth factor receptor
EMA: European Medicines Agency
gpNMB: Glycoprotein-NMB
IMMU-132: Sacituzumab govitecan
SGN-LIV1A: Ladiratuzumab vedotin
MMAE: Monomethyl-auristatin-E
ORR: Overall response rate
OS: Overall survival
PFS: Progression-free survival
CDX-011: Glembatumumab vedotin
CTA: Cancer-testis antigen
WT1: Wilms tumor protein 1
MAGE-12: Melanoma antigen gene-12
FRα: Folate receptor alpha
TAC: Tumor-associated carbohydrate
MSC: Mesenchymal stem cell
ILC: Invasive lobular carcinoma
IDC: Invasive ductal carcinoma
P4H: Prolyl 4-hydroxylase
NG2: Chondroitin sulfate proteoglycan
PDGFR: Platelet-derived growth factor receptor
FAP: Fibroblast activation protein
PDPN: Podoplanin
FSP: Fibroblast specific protein
ER: Estrogen receptor
myoCAF: Myofibroblastic CAF
iCAF: Inflammatory CAF
apCAF: Antigen-presenting CAF
α-SMA: α-Smooth muscle actin
CAV1: Caveolin 1
STAT3: Signal transducer and activator of transcription 3
CAP: Cancer-associated pericyte
NO: Nitric oxide
TGF: Transforming growth factor
LRRC15: Leucine-rich repeat-containing protein 15
PDAC: Pancreatic ductal adenocarcinoma
CXC: Cysteine X cysteine
TNF: Tumor necrosis factor
IFN: Interferon
IL: Interleukin
FASL: Fas ligand
BGN: Biglycan
ARG: Arginase
iNOS: Inducible Nitric oxide synthase
MHC: Major histocompatibility complex
JAK: Janus kinase
PI3K: Phosphatidylinositol-3 kinase
AKT: Protein kinase B
PG: Prostaglandin
COX2: Cyclooxygenase-2
PEG2: Prostaglandin E2
VEGF: Vascular endothelial growth factor
SPP1: Secreted phosphoprotein 1
BM-MSC: Bone marrow-derived mesenchymal stem cell
MZF1: Myeloid zinc finger 1
ADF: Adipocyte-derived fibroblast
MCP1: Monocyte chemotactic protein 1
CHI3L1: Chitinase-3-like protein 1
LAM: Lipid-associated macrophages
EMT: Epithelial-to-mesenchymal transition
SDF1: Stromal cell-derived factor 1
G-CSF: Granulocyte colony-stimulating factor
Breg: Regulatory B cell
HIF1α: Hypoxia-induced factor 1α
PDK1: 3-Phosphoinositide-dependent kinase 1
TCA: Tricarboxylic acid
LDHA: Lactate dehydrogenase A
PLAC8: Placenta-specific 8
UFM1: Ubiquitin-fold modifier 1
BATF: Basic leucine zipper ATF-like transcription factor
CRG: Cuproptosis-related gene
AIR: Acquired immune resistance
GEMM: Genetically engineered mouse model
DrIM: Disease related immune gene
NOD: Non-obese diabetic
MKO: Mouse knockout
ATAC: Assay for transposase accessible chromatin
TCR: T cell receptor
TAA: Tumor-associated antigen
HSP: Heat shock protein
CDK: Cyclin-dependent kinase
MUC4β: Mucin 4β
MSA2: Mammary serum antigen 2
TIL: Tumor-infiltrating lymphocyte
PET: Positron emission tomography
HR: Hormone receptor
NOX4: NADPH oxidase 4
FOLR: Folate receptor
TREM: Triggering receptor expressed on myeloid cell
ATRA: All-trans retinoid acid
ADAM12: A disintegrin and metalloproteinase-12
MCT1: Monocarboxylate transporter-1
CIAX: Carbonic anhydrase IX
PDT: Photodynamic therapy
ROS: Reactive oxygen species
DAMP: Damage-associated molecular pattern
MAF: Macrophage activating factor
PIT: Photoimmunotherapy
BP: Black phosphorus
BPNV: BP nanovesicles
APC: Antigen-presenting cell
NIR: Near-infrared
PTT: Photothermal therapy
BPQD: Black phosphorus quantum dots
